# Individuals with Major Depressive Disorder Report High Scores of Insecure-Avoidant and Insecure-Anxious Attachment Styles, Dissociative Identity Symptoms, and Adult Traumatic Events

**DOI:** 10.3390/healthcare9091169

**Published:** 2021-09-06

**Authors:** Sanobar Golshani, Ali Najafpour, Seyed Sepehr Hashemian, Nasser Goudarzi, Ali Firoozabadi, Mohammad Saeed Ghezelbash, Sara Hookari, Kimia Firoozabadi, Kenneth M. Dürsteler, Annette Beatrix Brühl, Mostafa Alikhani, Dena Sadeghi-Bahmani, Serge Brand

**Affiliations:** 1 Kermanshah University of Medical Sciences, Kermanshah 6719851115, Iran; snb.gln@gmail.com (S.G.); medicaldoctor91@gmail.com (M.S.G.); mostafaalikhani18@gmail.com (M.A.); 2 Student Research Committee, Faculty of Medicine, Kermanshah University of Medical Sciences, Kermanshah 6714869914, Iran; ali98aaa@yahoo.com; 3 Department of Psychology, Allameh Tabataba’i University, Tehran 1489684511, Iran; sepehr.hashemian@gmail.com; 4 Department of Psychiatry, AJA University of Medical Sciences, Tehran 1411718541, Iran; nassergoodarzi@yahoo.com; 5 Research Center for Psychiatry and Behavioral Sciences, Department of Psychiatry, Hafez Hospital, Shiraz University of Medical Sciences, Shiraz 7134814336, Iran; a_psych@hotmail.com; 6 Substance Abuse Prevention Research Center, Kermanshah University of Medical Sciences, Kermanshah 6714869914, Iran; sara_hoo64@yahoo.com (S.H.); dena.sadeghibahmani@upk.ch (D.S.-B.); 7 Faculty of Medical Sciences, UCL Medical School, University College London, London WC1E 6BT, UK; Kimiafiroozabadi65@gmail.com; 8 Division of Substance Use Disorders, Psychiatric Clinics, University of Basel, 4002 Basel, Switzerland; Kenneth.Duersteler@upk.ch; 9 Center for Addictive Disorders, Department of Psychiatry, Psychotherapy and Psychosomatics, Psychiatric Hospital, University of Zurich, 8001 Zurich, Switzerland; 10 Center for Affective-, Stress- and Sleep Disorders (ZASS), Psychiatric Clinics (UPK), University of Basel, 4002 Basel, Switzerland; annette.bruehl@upk.ch; 11 Faculty of Psychology, Stanford University, Stanford, CA 94305, USA; 12 Sleep Disturbances Research Center, Kermanshah University of Medical Sciences, Kermanshah 6719851115, Iran; 13 Department of Sport, Exercise, and Health, Division of Sport Science and Psychosocial Health, University of Basel, 4052 Basel, Switzerland; 14 Department of Psychiatry, School of Medicine, Tehran University of Medical Sciences, Tehran 1417466191, Iran

**Keywords:** major depressive disorder, dissociation, attachment style, childhood trauma, current traumatic events

## Abstract

Objective: Individuals with major depressive disorder (MDD) report more issues in social interaction compared to the general population. Moreover, dimensions of dissociation are considered dysfunctional strategies to cope with adverse life events. In this regard, current symptoms of MDD could be associated with traumatic events that occurred in childhood and in adult life. Given this background, the aim of the present study was to explore the associations between attachment styles as a proxy of quality of social interaction, dimensions of dissociation, and childhood and adult traumatic life events among individuals with MDD. Method: A total of 300 individuals with MDD (mean age: 31.31 years; 58.7% female) took part in this study. They completed a series of questionnaires on sociodemographic information, attachment styles, dimensions of dissociation, and childhood and adult traumatic life events. Results: Prevalence rates for attachment styles were as follows: anxious/ambivalent attachment style—71.7%; avoidant/dependent attachment style—13%; secure/close attachment style—15.3%. Compared to the general population, the participants reported higher prevalence rates of insecure attachment styles. Current symptoms of dissociation were associated with adult but not childhood traumatic life events. An anxious attachment style was associated with higher scores of dissociation. Conclusion: Psychotherapeutic treatment of individuals with MDD should consider the individuals’ challenging attachment styles and their risk of dissociation. While it is important to consider both adult and childhood traumatic events, in this research, more recent trauma occurring in adulthood was associated with current symptoms of dissociation.

## 1. Introduction

Major depressive disorder (MDD) is a severe and recurrent disease which affects people’s everyday functioning and quality of life [1]. Major depressive disorder is associated with a broad variety of both somatic and psychiatric comorbidity and mortality [2]. According to the DSM-5 [3] and others [4], the annual prevalence rate of MDD varies from 2.2% in Japan to 10.4% in Brazil; in Iran, the annual prevalence rate is about 4.1% [5]. Sociodemographic risk factors for MDD are the female gender [6,7] and being separated or divorced as a proxy of issues with social relationships [8]. Further risk factors include, but are not limited to, poor emotion regulation (see below), higher stress reactivity [6], poor sleep quality [9], a disbalance of neurotransmitters, including an increased cortisol secretion as a proxy of a deteriorated hypothalamus-pituitary-adrenocortical (HPA) axis activity [10], and subjectively perceived social rejection (see below).

In regard to the quality of social relationships, one’s ability to communicate closely with others appears to be one of the most important aspects of mental health. To illustrate, Hofstra et al. [11] investigated suicide incidence time trends in the Netherlands and observed a drop in incidences of suicide close to Christmas; such a drop could be understood as reflecting improved feelings of relatedness, thus reducing the tendency for individuals with suicidal ideation to attempt or to commit suicide. Ding et al. [12] observed that among individuals with MDD, perceived chronic social adversities appeared to trigger and maintain symptoms of stress, along with depression and anxiety. Individuals with MDD had more difficulties differentiating between faces displaying positive emotions and those showing no emotional content [13]. Relatedly, among individuals with MDD, emotion regulation and social interactions also appeared to be closely related. Visted et al. [14] concluded in their systematic review and meta-analysis that people with depressive episodes in their biographies (current or remitted state) reported having more difficulties with emotion regulation than people without such periods. More specifically, these difficulties in regulation appeared to persist even when symptoms of depression decreased. As a result, Visted et al. [14] speculated that poor emotion regulation and more limited social competencies might be latent risk factors for relapses. We took these observations into consideration in the following two ways: First, we introduced dimensions of dissociation as a proxy of dysfunctional emotion regulation. Second, we introduced attachment styles as a proxy of social competences.

Attachment styles are understood as a person’s tendency to seek closeness to a particular person and to feel more secure in her/his presence. Bowlby [15] was convinced that a child’s failure to form a secure attachment to one or more persons in the early years was related to an inability to develop close personal relationships in adulthood; as such, current attachment styles would mirror attachment styles developed during infancy [16]. Following Ainsworth and colleagues [17], three prototypical attachment style are identified: *secure, insecure-avoidant, insecure-ambivalent*. The key feature of the *secure* attachment style is full confidence and security in another person, which also enables an explorative behavior. The key feature of the *insecure-avoidant* attachment style is the behavior of avoiding close relationships with people, as close people might be perceived as a source of insecurity and disappointment. As such, adults with an *insecure-avoidant* attachment style eschew people in whom they could have confidence. The key feature of the *insecure-ambivalent* attachment style is the behavior of sometimes having full confidence in people one is close to, and at times avoiding them. Typically, adults with an *insecure-ambivalent* attachment style appear to oscillate between having confidence in another person and avoiding the same person, depending on the given context. Overall, it appears that adults with the *insecure-avoidant* and *insecure-ambivalent* attachment styles would have more difficulties in regulating their emotions and in building-up stable and long-term relationships with others. It follows that such individuals would have a higher risk of reporting mental distress, loneliness, and depression [18]. Furthermore, two recent studies [19,20] and two meta-analyses and systematic reviews [21,22] showed that the adult insecure attachment style was associated with a higher risk of MDD.

In the present study, we employed the Adult Attachment Scale [23] with the following attachment styles: *close subscale* (=*secure* attachment style); *dependence subscale* (=*insecure-avoidant* attachment style); *anxiety subscale* (=*insecure-ambivalent* attachment style). Given this background, we asked if and to what extent these attachment styles were associated with childhood and adult traumatic events, and with symptoms of dissociation as a proxy of a dysfunctional coping style. Furthermore, we assumed that compared to the general population [24], individuals with MDD would report more insecure attachment styles. More specifically, Berk [24] reported the following distributions of attachment styles in the general population: *secure* attachment style, 60–70%; *insecure-ambivalent/anxious* attachment style, 10–15%; *insecure-avoidant/dependent* attachment style, 10–15%.

There is research supporting the association between traumatic life events that occurred in childhood and current symptoms of dissociation and MDD; the following observations have been made so far: Three meta-analyses and systematic reviews [25,26,27] evidenced that adult MDD was associated with childhood abuse and childhood neglect as a proxy of childhood traumatic events. Compared to male adults, female adults had a higher risk of developing major depressive disorders after childhood abuse [26]. Compared to adults with no childhood abuse in their biography, adults with childhood traumatic events had a 2.5-fold higher risk of reporting MDD. Thus, childhood trauma appeared to be a major driver in the pathogenesis of MDD [6,28]. Relatedly, it appeared that traumatic childhood events unfavorably impacted the social interaction style among adults with MDD [29]. Given this background, we speculated that among individuals with MDD, reports of childhood traumatic events would be higher as compared to reports of adult traumatic events.

Lastly, in examining the associations between symptoms of dissociation in adulthood, symptoms of MDD, and childhood traumatic events, research is scarce: Cernis et al. [30] summarized in their systematic review and meta-analysis that the associations between symptoms of dissociation and symptoms of MDD were mixed and thus inconclusive. Others found in their systematic reviews and meta-analyses that childhood abuse and neglect as a proxy of childhood adversities increased the risk of symptoms of dissociation in adulthood [31,32]. Given this, we asked whether childhood and adult traumatic events were associated with symptoms of dissociations, along with current attachment styles.

The following four hypotheses and two research questions were formulated. First, we assumed that compared to the general population [24], individuals with MDD reported higher prevalence rates of insecure attachment styles. Second, following others [18,19,20,21,22], we assumed that individuals with MDD would report higher prevalence rates of insecure attachment styles as compared to a secure attachment style. Third, following previous research [31,32], we assumed that symptoms of dissociation would be associated with a higher prevalence of traumatic events. Fourth, following Negele et al. [29], we assumed that prevalence rates of childhood traumatic events would be higher as compared to adult traumatic events. The first exploratory research question asked if and to what extent current attachment styles and categories of dissociation did systematically modify scores of childhood and adult traumatic events. The second research question asked if attachment styles and categories of dissociation were associated.

We hold that the answers to these hypotheses and to these research questions are of practical and clinical importance, because the answers might have further impact on the treatment strategy. To this end, we assessed a larger sample of outpatients with diagnosed MDD.

## 2. Methods

### 2.1. Procedure

Outpatients with MDD of the Farabi Hospital in Kermanshah (Kermanshah, Iran) were approached from March 2016 to February 2017 and asked to participate in the present cross-sectional study. Participants were fully informed about the aims of the study and the confidential data handling. Thereafter, they signed the written informed consent. Participants completed a booklet of questionnaires covering sociodemographic information, attachment styles, dissociative experiences, and childhood and adult traumatic experiences. The ethical committee of the Kermanshah University of Medical Science (KUMS; Kermanshah, Iran; code: 96437) approved the study, which was performed in accordance with the rules established in the seventh and current form [33] of the Declaration of Helsinki.

### 2.2. Participants

The sample consisted of 300 outpatients with MDD. Inclusion criteria were: 1. Age between 18 and 65 years; 2. Diagnosis of MDD, as ascertained by trained and experienced psychiatrists and clinical psychologists, and based on the thorough clinical interview for DSM-5 diagnoses [34]; 3. Beck Depression Inventory [35,36], with scores between 19 and 29 points indicating a moderate self-reported MDD; 4. Willing and able to comply with the study conditions; 5. Signed written informed consent. Exclusion criteria were: 1. Current state of psychosis; 2. Current state of suicidality; 3. Severe MDD, as ascertained by trained and experienced psychiatrists and clinical psychologists, and based on a thorough clinical interview for DSM-5 diagnoses [34]; 4. Beck Depression Inventory [35,36], with scores of 30 points and higher indicating a severe self-reported MDD; 5. Prevalence of other psychiatric comorbidities such as bipolar disorder, post-traumatic stress disorder, substance use disorder, anxiety disorder, personality disorders or psychotic disorders, always based on a thorough clinical interview for DSM-5 diagnoses [34]; 6. Current treatment of electro-convulsive therapy (ECT); 7. Withdrawal from the study.

The intake of psychopharmacological medications was not an exclusion criterion. Out of the 350 individuals approached, 23 (6.6%) did not meet the inclusion criteria, and 27 (7.7%) refused to participate. The final sample size was 300.

### 2.3. Measures

#### 2.3.1. Sociodemographic Information

Participants reported their sex (female, male), age (years), civil status (single, married, divorced, widowed), employment (student/unemployed, self-employed, employed, housewife), and highest educational level (under diploma, high school diploma, bachelor’s degree, and higher).

#### 2.3.2. Adult Attachment Style

To assess attachment styles, the Farsi version [37] of the Adult Attachment Scale [23] was employed. It consists of 18 items; answers are given to 5-graded Likert scales ranging from 1 (=not at all my characteristics) to 5 (=entirely my characteristics), with higher scores reflecting a more pronounced attachment style. The following subscales were calculated: *anxiety subscale* (A; that is, *insecure attachment style* sensu Ainsworth et al. [17]); *close sub-scale* (C; that is, *secure attachment style* sensu Ainsworth et al. [17]); and *dependence sub-scale* (D; that is, *avoiding attachment style* sensu Ainsworth et al. [17]; Cronbach’s alpha: 0.79).

#### 2.3.3. Dissociative Experiences—Dissociative Experiences Scale (DES)

The Dissociative Experiences Scale (DES) is a questionnaire to self-assess dissociative experiences [38]. The Farsi version [39] had satisfactory psychometric properties. The questionnaire consists of 28 items. Typical items are: “I have had this experience that I have felt that other people, other objects, and the world around me are not real.”; “I have had that experience so I engaged with a fantasy as if that fantasy really happened to me.”. Answers are given on 10-point rating scales, with the anchor points ranging from 0% (never) to 100% (always); higher mean scores reflect a more pronounced experience of dissociation. The scores range from 0 to 100. The scale was used both as continuous and categorical variables with the following categories: 0–20 points, low dissociative experiences; 20–40 points, moderate dissociative experiences; 41 points and higher, severe dissociative experiences. This last category is equivalent to the DSM-5 categories of possible Posttraumatic Stress Disorder (PTSD), Dissociative Disorder Not Otherwise Specified (DDNOS), or Dissociative Identity Disorder (DID; Cronbach’s alpha: 0.82).

#### 2.3.4. Traumatic Events

The Farsi version [40] of the Traumatic Experience Checklist [41] was employed to assess childhood and adult traumatic events. It consists of 13 items; six items refer to traumatic experiences during childhood and adolescence (e.g., “Sexual contact before you were 18 with somebody who was 5 or more years older than you”; “life-threatening illness”); seven items refer to recent or traumatic events during adulthood (e.g., “imprisonment”; “torture”; “sexual assault by a family member or someone you know”). Answers are given in a forced-choice dichotomous fashion (no = 0; yes = 1). The following sum scores were calculated: childhood trauma; adult trauma; overall trauma score, with higher sum scores reflecting greater frequency of traumatic events (Cronbach’s alphas: from 0.83 to 0.89).

### 2.4. Statistical Analysis

The inspection with a series of Kolmogorov–Smirnov tests showed that outcome variables were normally distributed.

A series of Pearson’s correlations was performed to calculate the associations between age, dissociative dimensions, and traumatic events (always continuous variables).

An X^2^ test was performed to compare the current distributions of attachment styles with the distributions of attachment styles as expected in the general population [24].

A Bayes factor for one-samples was performed to calculate the distribution of secure/close versus insecure attachment styles.

A series of X^2^ tests was performed to calculate the distributions between adult attachment styles, categories of dissociative experiences, and gender, civil status, employment status, and educational level.

A *t*-test was performed to compare the means between childhood and adulthood traumatic events.

A multivariate ANOVA was performed with the following factors: adult attachment style (anxiety/insecure attachment style; close/secure attachment style; dependence/avoidant attachment style) and categories of dissociation (low, moderate, severe dissociation). Independent variables were: traumatic events such as childhood trauma, current trauma; overall score. Effect sizes were reported as partial eta squared.

An X^2^ test was performed to compare the current distributions of attachment styles with the categories of dissociation.

The level of significance was set at alpha < 0.05. All statistical computations were performed with SPSS^®^ 25.0 (IBM Corporation, Armonk, NY, USA) for Apple Mac^®^.

## 3. Results

### 3.1. Participants’ General Information

As shown in Table 1, a total of 300 individuals (outpatients) with MDD (58.7% females) took part in the study. Their mean age was 31.31 years (SD = 11.02). Participants were either married (51.3%), single (46.93%), or divorced (2.3%). In terms of employment status, participants were either students or unemployed (36.7%), self-employed (24%), employed (21.3%), or housewives (18%). Participants’ levels of education ranged from high school diploma and college diploma (45.7%) to a bachelor’s degree and higher (24.7%).

Forty-six (15.3%) reported a close/secure attachment style, and 254 (84.4%) reported an insecure attachment style; among those, 215 (71.4%) reported an anxious/ambivalent insecure attachment style, and 39 (13%) reported a dependent/avoidant insecure attachment style.

The categories of dissociation were as follows: 23 (7.7%) reported low dissociation, 171 (56.8%) reported moderate, and 106 (35.2%) reported severe dissociation. Note that the category of severe dissociation was equivalent to the DSM-5 categories of possible PTSD, Dissociative Disorder Not Otherwise Specified (DDNOS), or Dissociative Identity Disorder (DID).

Participants’ report of traumatic events included the following: childhood traumatic events had 5.82 points, adulthood traumatic events had 6.86 points, and the overall score was 12.68 points.

### 3.2. Adult Attachment Styles, Categories of Dissociative Experiences, and Sociodemographic Information

Attachment styles were unrelated to gender (X^2^ (*n* = 300, df = 2) = 2.39), civil status (X^2^ (*n* = 300, df = 4) = 2.17), the highest educational degree (X^2^ (*n* = 300, df = 3) = 2.34), and employment status (X^2^ (*n* = 300, df = 4) = 1.56; all ps > 0.40).

### 3.3. Comparisons of Attachment Styles between the Present Sample of Individuals with Major Depressive Disorders (MDD) and the General Population

Among individuals with MDD, the following attachment styles were observed: 46 (15.3%) reported a close/secure attachment style, and 254 (84.4%) reported an insecure attachment style; among those, 215 (71.4%) reported an anxious/ambivalent, and 39 (13%) reported a dependent/avoidant insecure attachment style. Berk [24] reported the following distributions of attachment styles in the general population: secure attachment style, 60–70% (calculated value: 70%; *n* = 230); insecure-ambivalent/anxious attachment style, 10–15% (calculated value: 15%; *n* = 35); insecure-avoidant/dependent attachment style, 10–15% (calculated value: 15%; *n* = 35). The X^2^ test was (*n* = 600; df = 3) = 236.88, *p* < 0.001. Compared to the general population, individuals with MDD reported a lower prevalence rate of a close/secure attachment style (*n* = 46), and higher prevalence rates of the insecure-anxious (*n* = 215) and insecure-avoidant/dependent (*n* = 39) attachment styles.

### 3.4. Distribution of Secure/Close versus Insecure (Dependent/Avoidant; Anxious/Ambivalent) Attachment Styles

The Bayes Factor test revealed a statistically and significantly lower score for the close/secure attachment style versus the insecure (dependent/avoidant; anxious/ambivalent) attachment styles (*n* = 300; M = 1.97, SD = 0.95; Bayes Factor: 0.000; t(299) = 51.12, *p* < 0.001).

### 3.5. Correlations between Age, Dissociative Dimensions, and Traumatic Events

Table 2 provides the associations between age, dissociative dimensions, and traumatic events; note that statistical indices are not reported in the text once again.

Higher age was associated with a higher score of traumatic events in adulthood (modest correlation). Age was not associated with dissociative dimensions, traumatic events in childhood, and the overall score of traumatic events.

Higher dissociative dimensions were modestly associated with a higher traumatic overall score.

Higher childhood traumatic events were associated with a higher adulthood traumatic event score and with a higher overall traumatic event score.

A higher adulthood traumatic event score was associated with a higher overall traumatic event score.

### 3.6. Childhood and Adulthood Traumatic Events

Participants reported statistically higher adulthood traumatic events (M = 6.86; SD = 0.47), compared to childhood traumatic events (M = 5.82; SD = 0.60; t(299) = 27.99, *p* < 0.001; d = 0.81; large effect size).

### 3.7. Categories of Dissociative Experiences and Impact of Adult Attachment Styles on Childhood Trauma, Adult Trauma, and Overall Trauma as a Function of Dissociation Categories and Attachment Styles (Categories)

A multivariate ANOVA with the factors of dissociation (low, moderate, severe dissociation), attachment style (anxiety/insecure; close/secure; dependent/avoidant), and the dependent variables of trauma (childhood; current; overall score) was performed. All F’s were <1.76, p’s > 0.17, partial eta-squared < 0.012, or simply put: the combination of dissociation categories and adult attachment styles did not systematically impact the scores of trauma (childhood, adult; overall score).

### 3.8. Associations between Dissociative Experiences and Adult Attachment Styles (Categorical Variables)

Table 3 provides the overview of frequencies and distributions between dissociative experiences (no, moderate, severe) and adult attachment styles (close, anxious, dependent).

There was a statistically significant difference of distributions between dissociative experiences and adult attachment styles (X^2^ (*n* = 300, df = 4) = 15.10, *p* < 0.01), that is to say:

Low dissociative experiences were associated with a higher frequency of a close/secure and dependent attachment styles, but a lower frequency of an anxious adult attachment style.

Moderate dissociative experiences were associated with a higher frequency of a close/secure attachment style, and a lower frequency of an anxious adult attachment style.

Severe dissociative experiences were associated with a higher frequency of an anxious adult attachment style, and a lower frequency of a close adult attachment style.

## 4. Discussion

The key findings of the present study demonstrate that compared to the general population, individuals with major depressive disorder (MDD) reported a lower prevalence rate of a secure/close attachment style, and higher prevalence rates of insecure-anxious/ambivalent and of insecure dependent/avoidant attachment styles. Furthermore, in the present sample, an insecure attachment style prevailed over a secure attachment style. A low dissociation category was also associated with a secure attachment style, while a severe dissociation category was associated with an anxious/ambivalent attachment style; however, the pattern of results was not that straightforward. In addition, categories of attachment styles and dissociation were unable to systematically impact the levels of childhood and adult traumatic events. Lastly, participants reported higher adult traumatic events as compared to childhood traumatic events. The present data expanded on the current knowledge in the field in the following three ways: First, we provided statistical evidence that compared to the general population, individuals with MDD reported more insecure attachment styles. Second, such attachment styles were associated with levels of dissociation. Third, the combination of dissociation categories and attachment styles did not systematically modify scores of childhood and adult traumatic events. And fourth, adult traumatic events were higher as compared to childhood traumatic events. We hold that the present results are of clinical and practical importance. First, when treating individuals with MDD, their attachment styles as a proxy of their social interactional style might challenge the therapeutic alliance and therapeutic relationship. Second, such demanding interactional styles might further be trialed by the presence of dissociation as a proxy of a dysfunctional coping strategy. Third, against expectations, adult traumatic events were more prevalent compared to childhood traumatic events, emphasizing that the treatment of adult and current life issues might be a more appropriate strategy as compared to that which focuses on childhood.

Four hypotheses and two research questions were formulated and each of these is now considered in turn.

With the first hypothesis, we assumed that compared to the general population [24], individuals with MDD would report higher prevalence rates of insecure attachment styles, and our data did confirm this. The novelty of the result is that we statistically compared and confirmed this assumption.

With the second hypothesis, we assumed that individuals with MDD would report more insecure attachment styles as compared to the secure/close attachment style, and again, the data confirmed this. Accordingly, the pattern of results replicates previous results [18,19,20,21,22]. In our opinion, the novelty of the study is that we gathered data from 300 individuals with MDD. As a comparison, Fuhr et al. [20] assessed 122 individuals with remitted MDD and remitted bipolar disorders; Picardi et al. [19] assessed 21 individuals with MDD and with bipolar disorders. The number of participants mentioned in the meta-analysis of Cortés-Garcia et al. [21] ranged from 52 to 2244, up to 7290 participants; however, such studies with a high sample size collected data exclusively from self-rating questionnaires, without providing a thorough clinical and expert-based assessment. As such, we claim that the present sample size is large, particularly when taking into consideration that trained and experienced psychiatrists and psychologists thoroughly interviewed and diagnosed all participants.

With the third hypothesis, we assumed that symptoms of dissociation would be associated with higher traumatic events, although data did not fully confirm this. As shown in Table 3, correlation coefficients between dissociative dimensions and traumatic events were spurious. The correlation coefficient between dissociative dimensions and the overall score of traumatic events was statistically significant, although the significant *p*-value was due to the large sample size [42,43,44,45,46]. As such, the present data do not match previous findings [31,32]. The quality of the data does not allow for a deeper understanding of the almost-zero result. Given this, we claim the following admittedly speculative assumptions: First, the measurements were too coarse-grained to allow for a detailed analysis of the data. Second, it is conceivable that the use of different assessment measurements did not allow for a comparison between and within the study results. Third, it is possible that further unassessed dimensions blurred the overall pattern of results. Fourth, it is conceivable that some participants showed very high correlation coefficients, while other participants did not; summing up their correlation coefficients resulted in this present zero result. Fourth, it is conceivable that some studies used categories of dissociation dimension; however, categorizing continuous data bears the risk of decreasing the quality of the data [47]. Fifth, there is really no association between dimensions of dissociation and traumatic events.

With the fourth hypothesis, we assumed that the prevalence rates of childhood traumatic events would be higher as compared to adult traumatic events; however, data did not confirm this. Accordingly, the present results are at odds with previous studies [29]. Once again, the quality of the data does not allow for deeper introspection into the underlying psychological mechanisms; speculatively, it is possible that processes of dissociation precluded the retrieval of information from long-term episodic memory. Given this, results suggest not to overestimate the experiences had in the past during childhood, but to at least equally focus on more recent adverse experiences in adulthood.

The first exploratory research question asked if current attachment styles and categories of dissociation systematically modified scores of childhood and adult traumatic events, and the answer was no. As such, the answer to this research question confirmed what has already been confirmed before—that attachment styles, categories of dissociation, and traumatic events in childhood and adulthood were not associated. Again, we claim that this result is intriguing, because it does not match and thus challenges previous study findings (see studies mentioned above).

The second research question asked if attachment styles and categories of dissociation were associated, and the overall answer was yes. Low dissociative experiences were associated with a higher frequency of a close/secure attachment style, but also of a dependent attachment style, and a lower frequency of an anxious adult attachment style. In contrast, severe dissociative experiences were associated with a higher frequency of an anxious adult attachment style and a lower frequency of a close adult attachment style.

Despite the novelty of the results, the following limitations should be considered. First, the cross-sectional nature of the study does not allow for a thorough identification of causality. This holds particularly true, as data from the traumatic event questionnaires may suggest causalities over time. However, retrospectively completing a questionnaire on childhood experiences always bears the risk of a memory and recall bias. As such, the overall pattern of results should be interpreted with caution.

Second, we assessed a sample of outpatient individuals with MDD of moderate severity; as such, the present results may not be transferred to individuals with severe MDD and/or currently treated as inpatients.

Third, it is possible that further latent and unassessed psychosocial dimensions could have biased two or more variables in the same or opposite direction.

Fourth, it might be questionable if and to what extent attachment styles acquired during early childhood should persist over time and cross into adulthood. If so, this would imply that a person would grow up in quite a stable psychosocial environment from (early) childhood to adulthood. This would also further imply that a person would be rather insensitive to social and psychosocial changes and stimulations. In this regard, others [19,20] claimed that an unfavorable attachment style such as an insecure-avoidant and insecure-ambivalent, acquired during an early stage of infancy, bears the risk of developing symptoms of depression. However, the opposite process is also observed. Individuals with symptoms of MDD withdraw from social activities, as social activities appear not to be satisfying and rewarding anymore. On the other hand, for instance, Hofstra et al. [11] observed a drop in incidences of suicides close to Christmas; such a drop was understood as reflecting improved feelings of relatedness, thus reducing the tendency for individuals with suicidal ideation to attempt or to commit suicide. As such, this change of suicidal behavior could be understood as an individual’s flexible adaptation to social realities. Relatedly, Ding et al. [12] observed that among individuals with MDD, perceived chronic social adversities appeared to trigger and maintain symptoms of stress, along with depression and anxiety. Similarly, among individuals with MDD, it also appeared that emotion regulation and social interactions are closely related. Visted et al. [14] concluded in their systematic review and meta-analysis that people with depressive episodes in their biographies (current or remitted state) reported having more difficulties with emotion regulation than people without such periods. More specifically, these difficulties in regulation appeared to persist even when symptoms of depression decreased. As a result, Visted et al. [14] speculated that poor emotion regulation and more limited social competencies might be latent risk factors for relapses. Given this background, it is conceivable that it is not the attachment styles, but the situational dysfunctional emotion regulation and the situational dysfunctional information processing of social signals that could be the main drivers of individuals’ behavior in social contexts.

Fifth, and as data showed, adult traumatic events appeared to have a higher impact on current psychosocial states as compared to childhood traumatic events.

Sixth, a major issue of the concept of attachment styles derives from the key study itself. That is to say, that the procedure of Ainsworth et al.’s [17] so-called Strange Situation was criticized in three ways. First, not all babies fit within the proposed categories; as a result, Main and Solomon [48] proposed and introduced the fourth category of the disorganized attachment style. We note that this category was not considered in the Adult Attachment Scale [23]. Second, Ainsworth et al.’s [17] attachment classification was based not on the baby’s distress when the mother left the room, but on how the baby reacted when the mother came back. As such, and third, the Stranger Situation paradigm did not consider the baby’s temperament. Fourth, attachment styles differ between cultures and over time as a proxy of individual psychosocial development [49].

Seventh and last, the type, dosages, regularity, and impact on memory and recall processes of medications were not assessed.

## 5. Conclusions

Compared to the general population, individuals with major depressive disorders reported prevalently insecure attachment styles. An insecure attachment style was associated with a more severe category of dissociation. Compared to childhood traumatic events, adult traumatic events were more prevalent. Given this, it appears that the psychotherapeutic treatment of individuals with MDD should consider tendencies of dissociation, and focus on the individual’s social interactional styles [50] as a proxy of attachment style. Furthermore, the treatment of adverse events that occurred during adulthood should be a treatment priority.

## Figures and Tables

**Table 1:** Frequency of sociodemographic dimensions, attachment styles, and categories of dissociation.

Variables		Number of Participants	Prevalence (%)
Gender	Male	176	58.7
	Female	124	41.3
Age range (years)	18–25	176	58.7
	25–35	104	34.7
	35–45	113	37.7
	45–55	44	14.7
	55–65	23	7.7
Civil status	Single	16	5.3
	Married	139	46.3
	Divorced	154	51.3
	Widowed	7	2.3
Employment status	Student/unemployed	-	-
	Self-employed	110	36.7
	Employed	72	24
	Housewives	64	21.3
Educational status	Under diploma	54	18
	High school diploma/college diploma	89 137	29.7 45.7
	Bachelor’s degree and higher		
Attachment styles	Close	46	15.3
	Insecure	254	84.4
	Anxious	215	71.4
	Dependent	39	13.0
Dissociative experiences	<20 = low dissociation	23	7.7
	20–40 = moderate dissociation	171	56.8
	>40 = severe dissociation	106	35.2
		M (SD)	
Traumatic events	Childhood traumatic events	5.82 (0.60)	
	Adulthood traumatic events Overall score	6.86 (0.47) 12.68 (0.86)	-

**Table 2:** Correlation coefficients between age, dimensions of dissociation, and traumatic experiences (always continuous dimensions).

Dimensions	Age	Dissociative Dimensions	Traumatic Events; Childhood	Traumatic Events; Adulthood	Traumatic Events; Total Score
Age	-	0.09	−0.05	0.12 *	0.03
Dissociative dimensions		-	0.10	0.08	0.12 *
Traumatic events					
Childhood			-	0.29 ***	0.85 ***
Adulthood				-	0.75 ***
Total score					-

Notes: * = *p* < 0.05; *** = *p* < 0.001.

**Table 3:** Associations between categories of dissociation and attachment styles.

Adult Attachment Style	Dissociative Experiences; Categories			
	Low	Moderate	Severe	Total
Close/secure	4 (8.7%)	35 (76.1%)	7 (15.2%)	46 (100%)
Anxious/ambivalent	13 (6.0%)	114 (53.0%)	88 (40.9%)	215 (100%)
Dependent/avoidant	6 (15.4%)	22 (56.4%)	11 (28.2%)	39 (100%)
Total	23 (7.7%)	171 (57.0%)	106 (35.3%)	300 (100%)

## Data Availability

Data are made available upon request to experts in the field and upon through explanations of why and how data are used.
